# Uncovering hub genes in sepsis through bioinformatics analysis

**DOI:** 10.1097/MD.0000000000036237

**Published:** 2023-12-01

**Authors:** Zhao Liu, Eryue Qiu, Bihui Yang, Yiqian Zeng

**Affiliations:** a Department of Critical Care Medicine, Zhuzhou Central Hospital, Zhuzhou, China; b Department of Trauma Center, Zhuzhou Central Hospital, Zhuzhou, China; c Department of Hematology, Zhuzhou Central Hospital, Zhuzhou, China.

**Keywords:** bioinformatics analysis, biomarkers, diagnosis, sepsis

## Abstract

In-depth studies on the mechanisms of pathogenesis of sepsis and diagnostic biomarkers in the early stages may be the key to developing individualized and effective treatment strategies. This study aimed to identify sepsis-related hub genes and evaluate their diagnostic reliability. The gene expression profiles of GSE4607 and GSE131761 were obtained from the Gene Expression Omnibus. Differentially co-expressed genes between the sepsis and control groups were screened. Single-sample gene set enrichment analysis and gene set variation analysis were performed to investigate the biological functions of the hub genes. A receiver operating characteristic curve was used to evaluate diagnostic value. Datasets GSE154918 and GSE185263 were used as external validation datasets to verify the reliability of the hub genes. Four differentially co-expressed genes, FAM89A, FFAR3, G0S2, and FGF13, were extracted using a weighted gene co-expression network analysis and differential gene expression analysis methods. These 4 genes were upregulated in the sepsis group and were distinct from those in the controls. Moreover, the receiver operating characteristic curves of the 4 genes exhibited considerable diagnostic value in discriminating septic blood samples from those of the non-septic control group. The reliability and consistency of these 4 genes were externally validated. Single-sample gene set enrichment analysis and gene set variation analysis analyses indicated that the 4 hub genes were significantly correlated with the regulation of immunity and metabolism in sepsis. The identified FAM89A, FFAR3, G0S2, and FGF13 genes may help elucidate the molecular mechanisms underlying sepsis and drive the introduction of new biomarkers to advance diagnosis and treatment.

## 1. Introduction

Sepsis is a life-threatening organ dysfunction caused by a dysregulated host response to an infection.^[1]^ It remains a major cause of death in critical care units, and septic shock is a severe phase of sepsis.^[2]^ However, the precise pathogenesis of sepsis has not been elucidated. Early and accurate diagnosis and treatment of sepsis can suppress the disease progression. However, it usually lacks specific clinical symptoms, particularly in the early stages.^[3–5]^ Traditional biomarkers include white blood cell count and procalcitonin, interleukin-6, and C-reactive protein levels, all of which have limited specificity.^[6]^ Most did not show a clear advantage in assessing patient clinical outcomes. Thus, further in-depth studies on the mechanisms of pathogenesis and diagnostic biomarkers in the early stages may be the key to the development of individualized and effective treatment strategies. Some recent studies attempted to find reliable novel biomarkers for sepsis,^[7,8]^ for example, Monocyte distribution width seems to have the potential to be an early diagnostic marker.^[9–11]^ The rapid development of bioinformatics has provided a variety of methods to study the molecular mechanisms of sepsis and to search for novel biomarkers.^[12,13]^ This study aimed to identify sepsis-related hub genes and evaluate their diagnostic reliability. To obtain sepsis-related hub genes, we worked with the sepsis-related gene expression profile from the Gene Expression Omnibus databases using weighted gene co-expression network analysis (WGCNA) and differential gene expression analysis. The underlying pathogenesis of sepsis was further investigated using gene set variation analysis (GSVA), single-sample gene set enrichment analysis (ssGSEA), and receive operating characteristic (ROC) curve analysis.

## 2. Materials and methods

### 2.1. Microarray data

The normalized expression profiles of GSE4607 and GSE131761 were downloaded from the Gene Expression Omnibus (http://www.ncbi.nlm.nih.gov/gds) using the R package GEOquery. In GSE4607, the patients meeting the criteria for sepsis were enrolled in this study. Whole blood samples from 69 patients with septic shock and 15 controls were used for subsequent analysis. Moreover, whole blood-derived RNA transcriptome profiling was performed using the GPL570 platform. Peripheral blood sample data from 81 adult patients with postsurgical sepsis and 15 controls were obtained from GSE131761 for subsequent analysis. The probes were matched to gene symbols through the platform annotation files. Ethical approval was not necessary because this is a bioinformatics analysis based on previously public database.

### 2.2. Identification of key co-expression modules using WGCNA

We searched for co-expressed gene modules using WGCNA and investigated the correlation between gene networks and phenotypes, as well as the core genes in the network. In our study, the gene expression data of GSE4607 and GSE131761 were used to construct gene co-expression networks using the WGCNA package in R. To build a scale-free network, soft powers (β) were selected using the pickSoftThreshold function. The weighted adjacency matrix was transformed into a topological overlap matrix to estimate network connectivity. A hierarchical clustering method was used to construct the clustering tree structure of the topological overlap matrix. Different branch clusters represent distinct gene modules, and different colors represent different modules. Based on the weighted correlation coefficients, the genes were classified according to their expression patterns. Genes with similar expression patterns were grouped into modules, and thousands of genes were divided into multiple modules based on their gene expression patterns.

### 2.3. Differential expression analysis and interaction with the selected modules

To identify the differentially expressed genes (DEGs) among sepsis and control blood samples, the R “limma” package was applied in the GSE4607 and GSE131761 datasets. The P-values were corrected using the false discovery rate method. Genes that met the cutoff criteria of |logFC| ≥ 1.0 and *P* < .05 were screened out as DEGs. The results were presented on a volcano plot. Next, overlapping genes among the DEGs and co-expressed genes were analyzed using the R package VennDiagram. Then, the overlapping gene datasets from GSE4607 and GSE131761 were combined. Principal component analysis was used to confirm the corrected batch effects using the ComBat algorithm in the R “SVA” package.

### 2.4. Implementation of ssGSEA

The ssGSEA method was used to assess immune cell infiltration levels based on hub gene expression. We identified a set of marker genes associated with immune cell types, including different immune cells, immune-related pathways, and functions from a previous study.^[13]^ We applied ssGSEA to calculate the enrichment score of each immune-related term through the “GSVA” R package.^[14]^

### 2.5. Implementation of GSVA

GSVA is a non-parametric, unsupervised gene set enrichment method.^[14]^ GSVA was performed using the “GSVA” R package to score the gene sets of interest. Moreover, pathway-level gene expression changes were identified using gene set enrichment analysis to assess the biological functions of the candidate genes and different samples. The Molecular Signatures Database v7.0 was used to perform GSEA within the hallmark gene sets.

### 2.6. ROC analyses and comparison of the hub gene content in the 2 groups (sepsis and control groups)

To evaluate whether the hub genes had diagnostic value for sepsis, the content of hub genes in the 2 groups was presented as a box plot. Meanwhile, ROC analyses were performed using the R “pROC” package.^[15]^ The diagnostic accuracy of these genes for sepsis was evaluated using the area under the ROC curve values. If the area under the curve value was >0.8, the gene could be differentiated between patients with sepsis and controls.

### 2.7. External validation of the reliability of the hub genes

The GSE154918 and GSE185263 datasets were used as external validation datasets to verify the reliability of the hub genes. The expression levels of the hub genes in the healthy and sepsis groups in the 2 datasets were extracted and calculated.

### 2.8. Statistical analysis

Statistical analyses were performed using R version 3.6.3 (https://cran.r-project.org/bin/windows/base/old/3.6.3/). Differences in the distribution of categorical variables were compared using the chi-squared or Fisher exact tests. ROC curves were used to estimate the diagnostic value of the hub genes. Further, Pearson correlation coefficient was used for correlation analyses. Statistical significance was set at *P* values < .05.

## 3. Results

### 3.1. Construction of weighted gene co-expression modules

**Figure 1.**
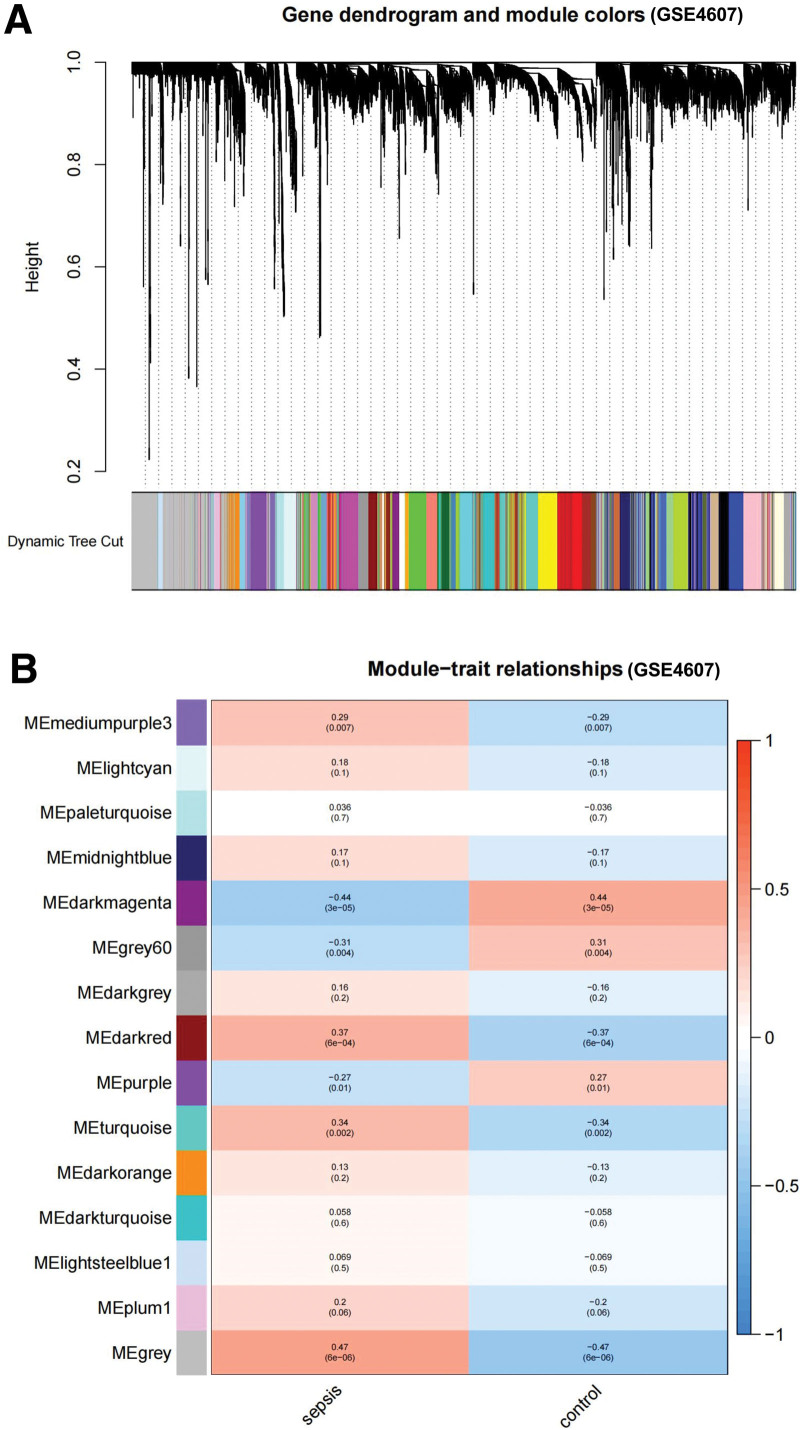
Identification of modules related to the clinical information in GSE4607 dataset. (A) The Cluster dendrogram of co-expression network modules. (B) Heatmap of module-trait relationships.

**Figure 2.**
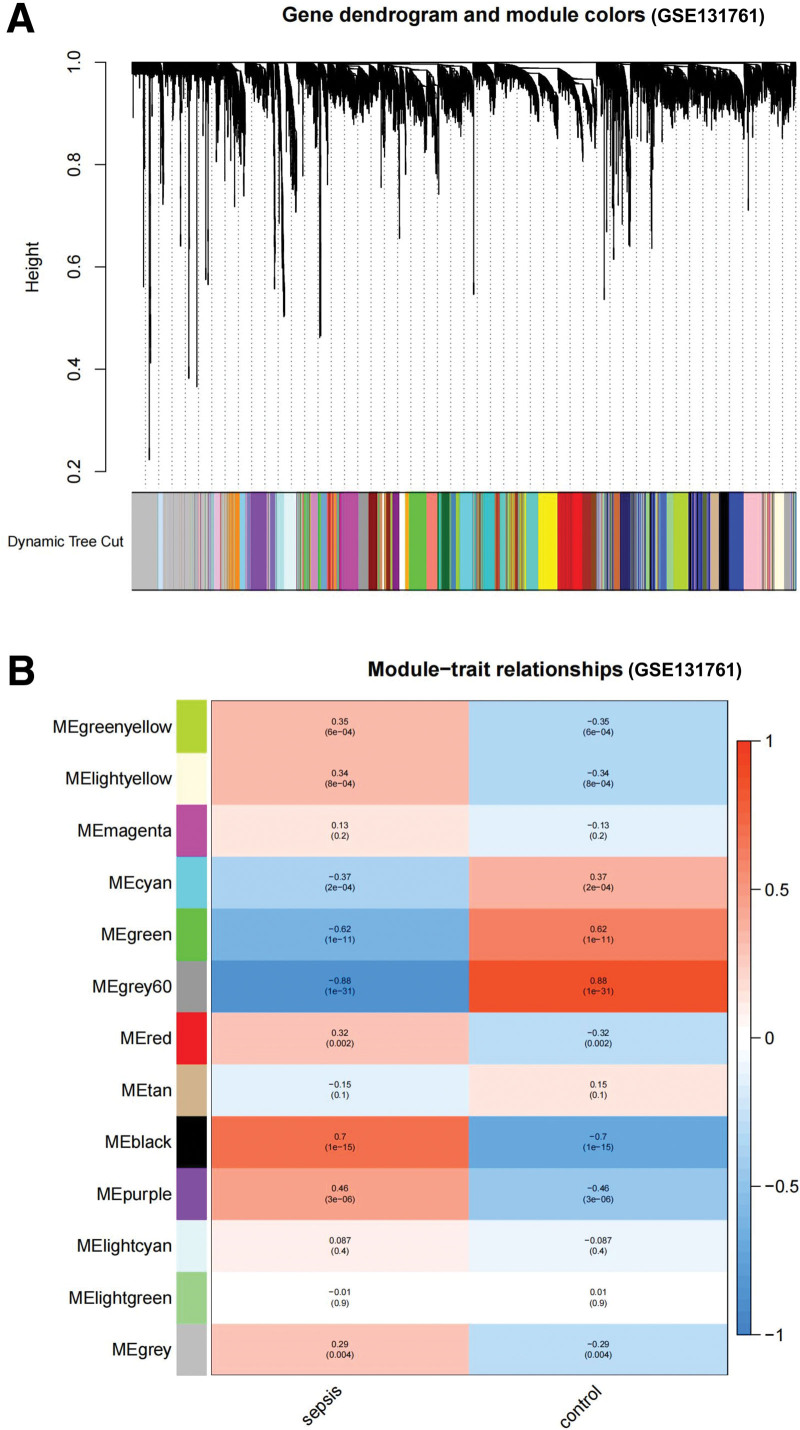
Identification of modules associated with clinical information in GSE131761 dataset. (A) The Cluster dendrogram of co-expression network modules. (B) Heatmap of Module-trait relationships.

Gene expression data from GSE4607 and GSE131761 were used to construct gene co-expression networks. After data preprocessing, the genes were screened for further analysis. In our study, soft powers (β) 18 and 8 were chosen for the soft-threshold parameter to ensure a scale-free network. Each module corresponded to one color. Fifteen modules in GSE4607 (Fig. 1) and 13 in GSE131761 (Fig. 2) were identified (excluding gray modules that were not classified into any clustering). Heatmaps of the module trait relationships were drawn, and correlations between the modules and clinical characteristics (sepsis and control groups) were assessed. The results are shown in Figure 1, indicating that the gray module in GSE4607 and the black module in GSE131761 had significant relationships with the sepsis group (gray module: *R* = 0.47, *P* = 6e–06; black module: *R* = 0.7, *P* = 1e–15).

### 3.2. Identification of genes between the DEG lists and co-expression modules

**Figure 3.**
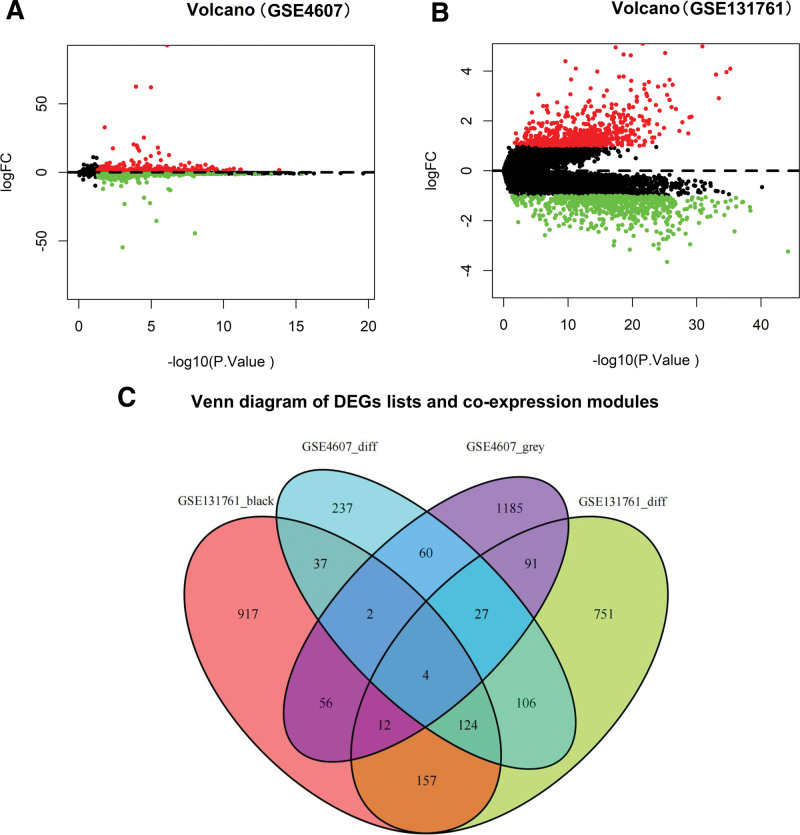
Identification of DEGs between GSE4607 and GSE131761 datasets of sepsis with the cutoff criteria of |logFC| ≥ 1.0 and *P* < .05. (A) Volcano plot of DEGs in the GSE4607 dataset. (B) Volcano plot of DEGs in the GSE131761 dataset. (C) Venn diagram showing 4 overlapping genes between DEGs and co-expression module (GSE4607_diff, DEGs of GSE4607; GSE4607_grey, co-expressed genes in the gray module of GSE4607; GSE131761_black, co-expressed genes in the black module of GSE131761; GSE131761_diff, DEGs of GSE131761). DEG = differentially expressed gene.

According to the cutoff criteria of |logFC| ≥ 1.0 and *P* < .05, 610 DEGs in the GSE4607 dataset (Fig. 3A) and 1272 in the GSE131761 dataset (Fig. 3B) were extracted in whole blood samples from patients with sepsis by “limma” package. As illustrated in Figure 3C, 93 and 297 co-expressed genes were extracted separately from the gray and black modules of the GSE4607 and GSE131761 datasets, respectively. Finally, we identified 4 overlapping genes, FAM88A, FFAR3, G0S2, and FGF23, which were extracted to explore further their function and diagnostic values (Fig. 3C).

### 3.3. Immune microenvironment landscape of sepsis

**Figure 4.**
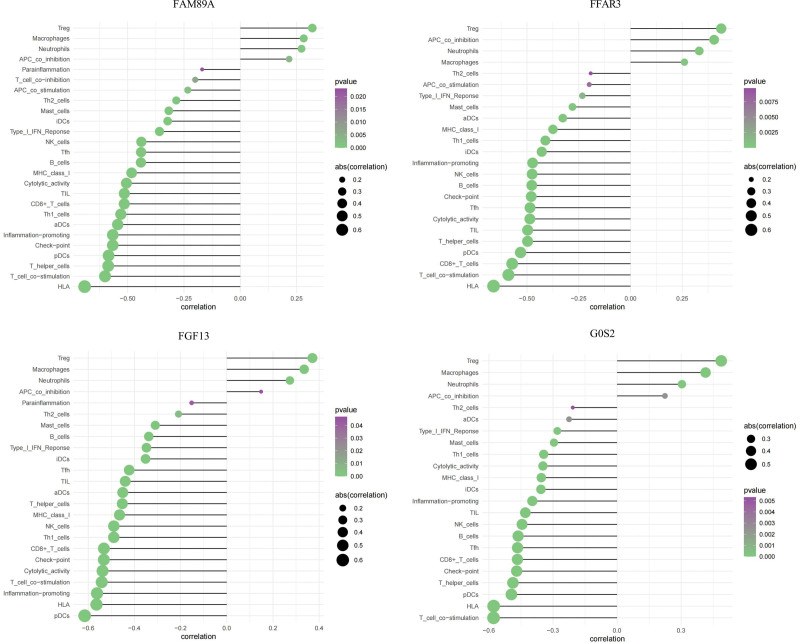
Pearson correlations of the 4 hub genes expression with the infiltration levels of various types of immune cells (APC = antigen-presenting cells, DC = dendritic cells, IFN = interferon, MHC = major histocompatibility complex, NK = Natural killer cells, Th = T helper cells, Treg = regulatory T cells).

The potential molecular mechanisms by which core genes influence disease progression were explored by analyzing the relationship between core genes and immune infiltration in the datasets. The results suggested that the 4 genes were significantly positively associated with Tregs, APC co-inhibition, and macrophages and were significantly negatively correlated with HLA, CD8+ T cells, and T cell co-stimulation. These 4 genes were significantly associated with the immune cell content (Fig. 4), and these results met our expectations.

### 3.4. GSVA analysis

**Figure 5.**
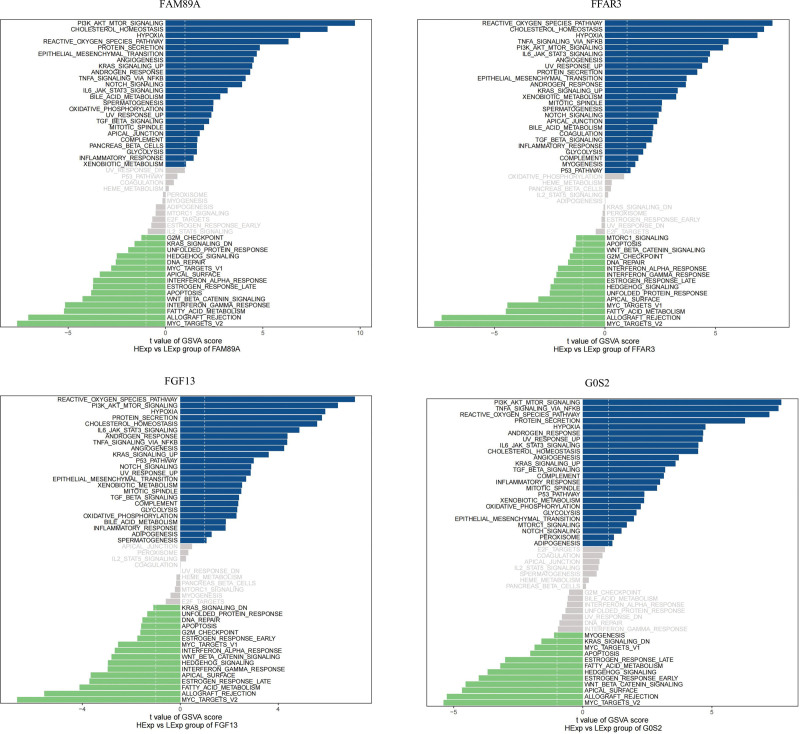
Differences in pathway activities of the 4 hub genes scored by GSVA between sepsis and control patients. *T* values were derived from a linear model. The cutoff value was set at |*t*| > 1. The blue column represents activated pathways while the green column represents the downregulation of pathways in sepsis patients (DN, down; UV, ultraviolet; v1, version 1; v2, version 2).

We explored the signaling pathways relevant to the 4 hub genes and analyzed the underlying molecular mechanisms associated with disease progression. The pathway analysis indicated that the 4 hub genes were related to multiple sepsis-related immune signaling and metabolic pathways (Fig. 5).

### 3.5. Box plot comparison of the hub genes content between sepsis and controls

**Figure 6.**
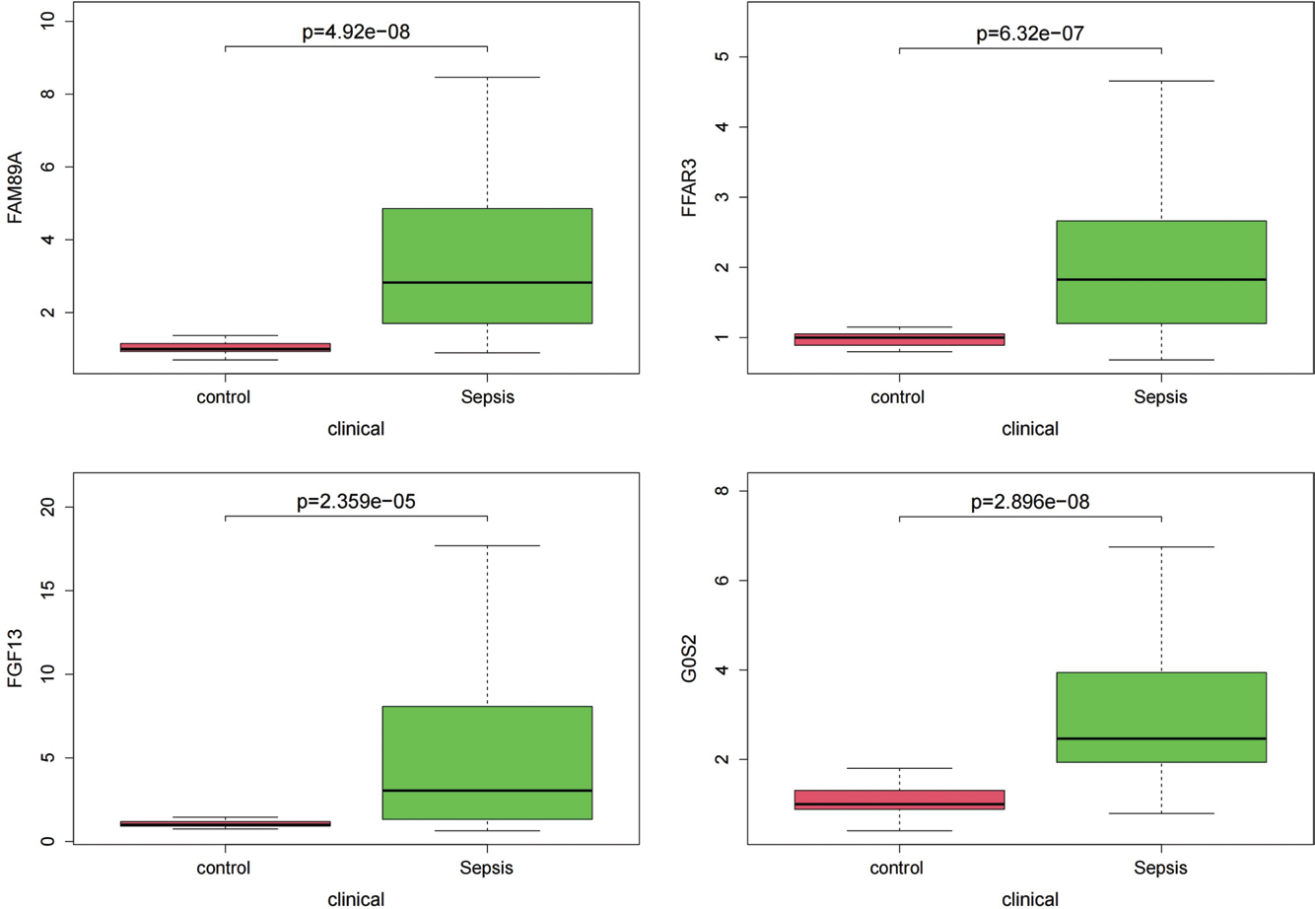
Box plot comparison of the 4 hub genes expression between sepsis and controls in GSE4607 dataset showed a significant difference among all the comparison groups.

**Figure 7.**
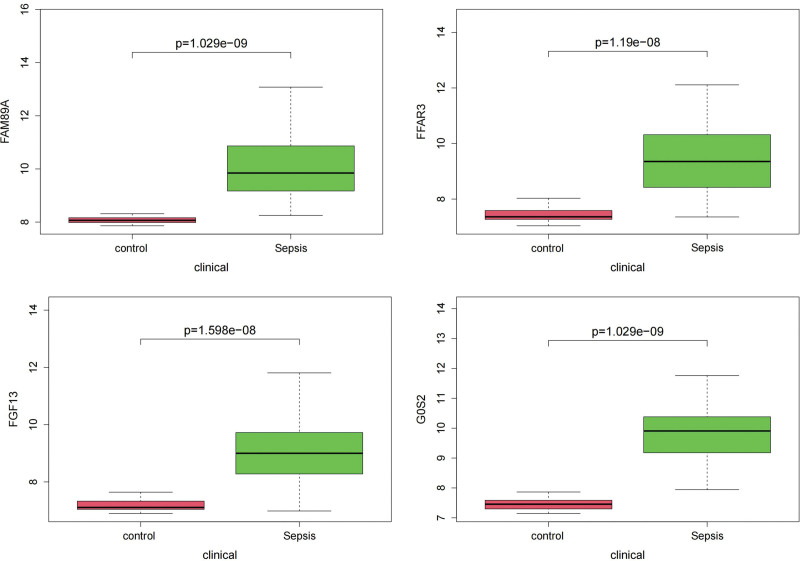
Box plot comparison of the 4 hub genes expression between sepsis and controls in GSE131761 dataset showed a significant difference among all the comparison groups.

As shown in Figures 6 and 7, the hub gene content was significantly different among all comparison groups.

### 3.6. ROC analysis

**Figure 8.**
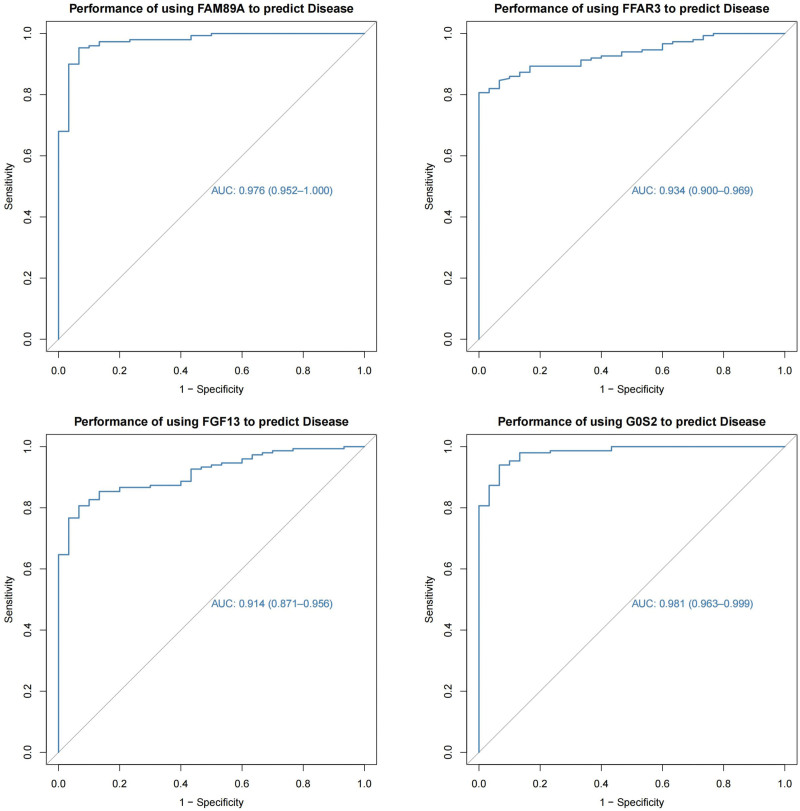
ROC curve of the 4 hub genes (FAM89A, FFAR3, FGF13, G0S2) to differentiate sepsis patients from controls. ROC = receive operating characteristic.

To further evaluate the value of these 4 hub genes in the diagnosis of sepsis, ROC curve analysis was performed. The results are presented in Figure 8. This indicated that the 4 genes could dramatically differentiate septic blood samples from non-septic control samples.

### 3.7. External validation of the 4 hub genes

**Figure 9.**
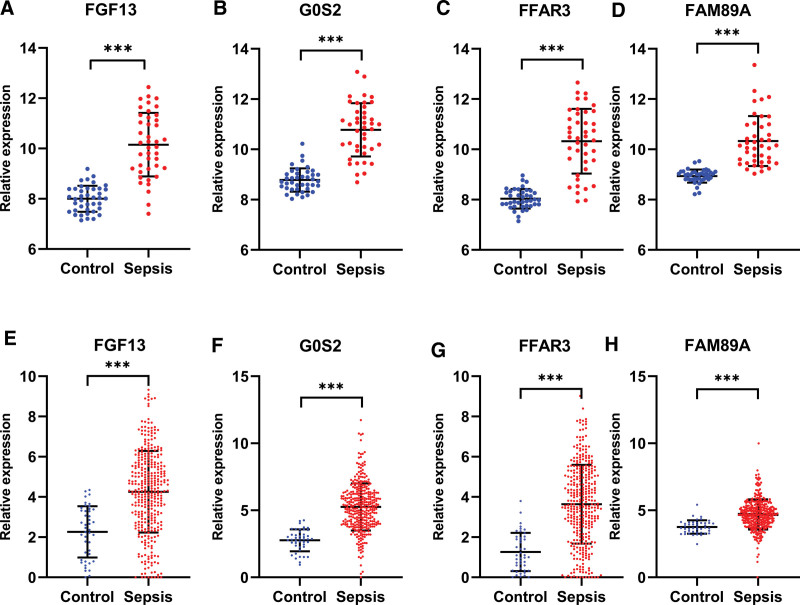
External validation of the 4 hub genes. (A–D) The relative expression of 4 hub genes between sepsis and controls in GSE154918 (***P < .001). (E–H) The relative expression of 4 hub genes between sepsis and controls in the GSE185263 (***P < .001).

**Figure 10.**
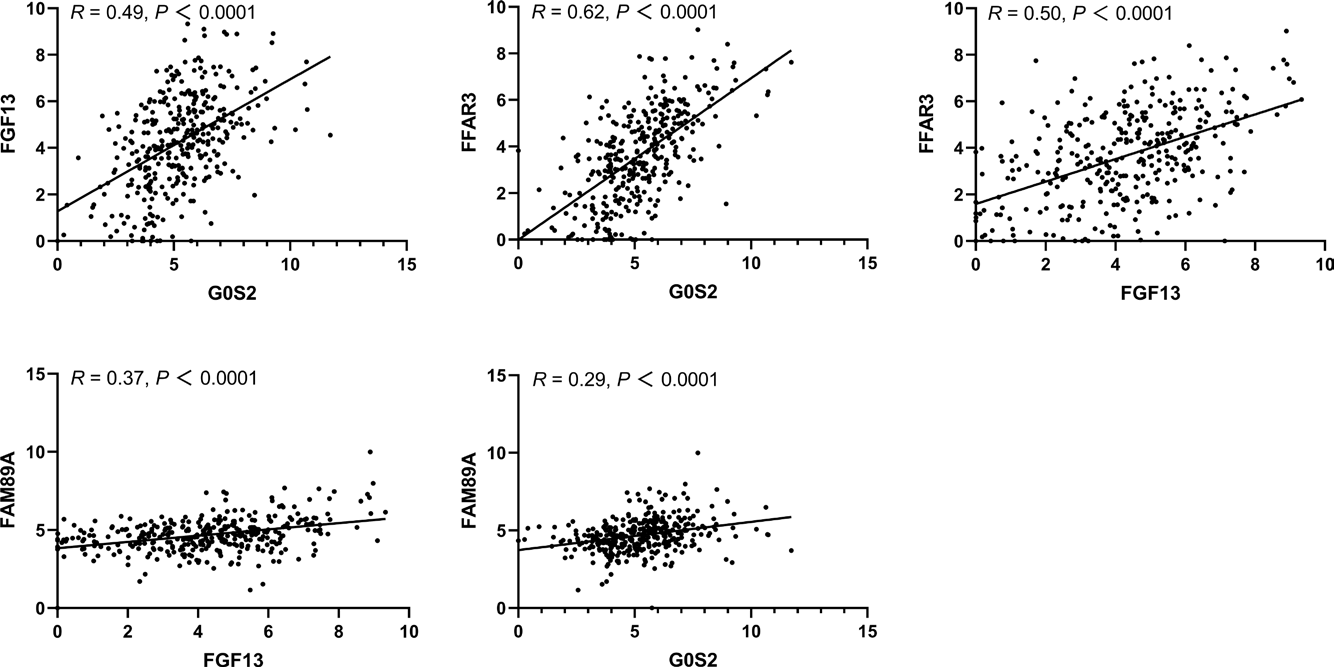
Pearson correlation analysis of the 4 genes in patients with sepsis in GSE185263 (***P < .001).

The GSE154918 dataset included 40 healthy controls and 42 patients with sepsis, whereas the GSE185263 dataset included 46 healthy controls and 302 patients with sepsis. The results showed that the relative expression of the 4 genes in the sepsis group was significantly higher than that in the healthy group in both datasets (Fig. 9). Pearson correlation analysis of the 4 genes in patients with sepsis using GSE185263 showed a moderate correlation between G0S2 and FFAR3 and between G0S2 and FGF13 (Fig. 10).

## 4. Discussion

Sepsis is a heterogeneous and complex syndrome that is induced by a dysregulated host response to an infection. The complexities of the mechanisms underlying sepsis pose significant challenges to researchers. Early studies on sepsis mainly focused on infection, but subsequent clinical studies indicated that the immune response plays a major role in sepsis.^[16]^ Some studies have suggested that acquired immune system dysregulation and metabolic disorders both play important roles in the process of sepsis.^[17–19]^

In this study, we performed bioinformatics analysis based on relevant microarray datasets to identify several potential hub genes of sepsis. The results suggest that FFAR3, FGF13, FAM89A, and G0S2 could significantly distinguish between septic blood samples and control samples. The hub gene content in all sepsis groups was higher than that in the non-sepsis control group. Furthermore, ROC analysis showed the diagnostic value of the peripheral blood levels of FAM89A, FFAR3, G0S2, and FGF13 in discriminating septic from non-septic control samples. The reliability of the 4 hub genes was confirmed using the GSE154918 and GSE185263 datasets. Therefore, we hypothesized that these 4 hub genes could play vital roles in the development and progression of sepsis.

Currently, there are only a few studies on the mechanisms underlying the 4 genes involved in the pathogenesis of sepsis. Previous studies elucidating the functions of these genes have been limited. FAM89A appears to be related to the immune response, and 2 previous studies indicated that the expression levels of the FAM89A gene could be used to differentiate bacterial from viral infections in the early phase, with a lower expression observed in viral infections and a higher expression observed in bacterial infections.^[20,21]^ Moreover, studies have found that the expression of FAM89A is increased in children with sepsis, which is consistent with our findings.^[22]^ However, the specific biological role of FAM89A in sepsis and its underlying mechanisms remain unclear.

FFAR3 is highly expressed in adipose tissues, pancreas, and neuronal cells, where it regulates metabolism.^[23]^ Furthermore, this gene is involved in the immune response, and its expression is also observed in the spleen, leukocytes, and immune cells involved in innate and adaptive immunity.^[24]^ FFAR3 is a G-protein-coupled receptor for short-chain fatty acids (SCFAs).^[25]^ SCFAs, the most abundant microbial metabolites in the intestine, have a strong effect on systemic innate cells and many types of DC- or macrophage-mediated immune responses.^[26]^ SCFA function is mediated by the FFAR3 receptors.^[27]^

G0S2 was first discovered in peripheral blood mononuclear cells and is related to the cell cycle.^[28]^ Research has revealed that G0S2 has an important role in cell inflammation and apoptosis.^[29]^ Moreover, a previous study showed that G0S2 is a positive regulator of oxidative phosphorylation, which increases mitochondrial ATP production, even under hypoxia.^[30]^

FGF13 is an important regulator of mitochondrial homeostasis in the endothelium in diabetic nephropathy.^[31]^ In addition, some studies have argued that FGF13 has important effects on abnormal glucose tolerance through the PI3K-AKT signaling pathway.^[32]^ A study also considered that FGF13 potentiates pathological cardiac hypertrophy via the activation of NF-κB.^[33]^

The present study confirmed that there was a significant positive correlation between FFAR3 and G0S2 and between FGF13 and G0S2. Collectively, these studies indicated that FFAR3, G0S2, and FGF13 play important roles in the regulation of inflammation, immunity, and metabolism. Research over the last few years suggests that uncontrolled inflammatory responses and metabolic reprogramming play important roles in sepsis and sepsis-associated organ dysfunction. Therefore, it appears likely that FFAR3, G0S2, and FGF13 participate in inflammation and metabolic reprogramming during sepsis.

Furthermore, ssGSEA and GSVA confirmed this hypothesis. ssGSEA suggested that some immune cells and functions were significantly enriched in FFAR3, FGF13, FAM89A, and G0S2. We found that the 4 hub genes were significantly positively correlated with Tregs, macrophages, and APC co-inhibition and significantly negatively correlated with HLA, CD8^+^ T cells, and T cell co-stimulation. Moreover, we discovered using GSVA that many signaling pathways, including the reactive oxygen species pathway, PI3K/AKT/mTOR signaling pathway, and hypoxia, were enriched in the 4 hub genes. These pathways have been demonstrated to play key roles in the regulation of inflammation and metabolism in sepsis.^[34–38]^

This study had some limitations. First, relevant experimental studies should be conducted to further confirm the biological roles of these genes and the signaling pathways involved in sepsis. Second, clinical information, such as survival time, could not be obtained, and the corresponding joint analysis with gene expression could not be performed to further evaluate the value of these genes.

In conclusion, we identified FFAR3, FGF13, FAM89A, and G0S2 as genes that can distinguish septic samples from non-septic samples. However, further studies are still required to verify the exact mechanism underlying sepsis development.

## Author contributions

**Conceptualization:** Zhao Liu, Yiqian Zeng.

**Data curation:** Zhao Liu, Eryue Qiu, Bihui Yang.

**Formal analysis:** Zhao Liu, Eryue Qiu, Bihui Yang, Yiqian Zeng.

**Funding acquisition:** Yiqian Zeng.

**Methodology:** Zhao Liu.

**Project administration:** Zhao Liu.

**Supervision:** Yiqian Zeng.

**Validation:** Eryue Qiu.

**Visualization:** Eryue Qiu, Bihui Yang.

**Writing – original draft:** Zhao Liu, Yiqian Zeng.

**Writing – review & editing:** Zhao Liu.
